# Dental Dispensaries

**Published:** 1899-09

**Authors:** 


### Dental Dispensaries.

A new movement that has been inaugurated in Chicago is perhaps worthy of a passing comment. Dispensary physicians are familiar with the fact that a very large proportion of those who depend on these institutions for medical treatment are in bad shape as regards their dental apparatus, and that this is true of the young as well as the older dispensary patrons. Dr. Carl T. Gramm (*Dental Review*, February) has called attention to these facts and made the proposition to establish dental dispensaries in the poorer districts of the city, where the class who at present are totally neglected from a dental point of view can be treated. Largely through his exertions the proposition has been carried into effect, and one or more of these dispensaries are already in operation. It is needless to say that the amount of good that can be done by a systematized effort in this direction is almost beyond calculation, and as it covers a class that at present pay no attention whatever to dental hygiene, it cannot seriously injure the practice of the dental profession, certainly not if carried out according to the plan laid down by Dr. Gramm. Recognizing the abuses in the medical dispensaries, he proposes to guard against them and to charge at least nominal fees and utilize all the aid of the Bureau of Associated Charities in distinguishing the worthy from the unworthy. Some impostors will be served, but the advantages from educating the poorer classes to care for their teeth will more than offset any loss the dental profession may suffer through them; and as Dr. Gramm says, the medical profession owes and performs its duty to the poor, and the dental profession should also do its duty within the bounds of its special sphere.—*Journal Am. Med. Assn.*

HERR METZGER, a surgeon-dentist of Chemnitz, in Saxony, is enthusiastic in his praise of indentol as a local anasthetic in cases of hypersensitive dentine. "It never leaves one entirely in the lurch if properly employed, but the operator most have sufficient patience to make three or more applications if necessary."

